# The urethral hanging theory and how it relates to Enhörning’s theory and the integral theory

**DOI:** 10.1007/s00192-019-04170-x

**Published:** 2019-12-17

**Authors:** Bo S. Bergström

**Affiliations:** 1 Years from 1988 to 2000, Chief of Department of Obstetrics & Gynecology Mora Hospital, 792 51 Mora, Sweden; 2 Years from 2000 to 2006, Overlege at Nordfjord Hospital, N-6771 Nordfjordeid, Norway; 3 Karlavägen 27, 11431 Stockholm, Sweden

**Keywords:** Stress urinary incontinence, Urgency, Urethral funneling, Pathophysiology, Mobility, TVT, TOT

## Abstract

**Introduction and hypothesis:**

The article discusses three theories of stress urinary incontinence, the urethral hanging theory, Enhörning’s theory, and the integral theory.

**Methods:**

The abdominal pressure transmission theory proposed by Enhörning is often misunderstood. It is regularly interpreted to mean that, in cases of stress urinary incontinence, the bladder neck descends outside the abdominal cavity, and treatment must involve elevating or repositioning the bladder neck.

**Results:**

However, this actually contradicts the information provided in Enhörning’s original paper. The urethral hanging theory accepts the core of Enhörning’s theory and the integral theory rejects it. The three theories have different views on closure and opening of the bladder neck and on the pathophysiology of urethral funneling.

**Conclusion:**

These differences are described and discussed.

## Discussion

**Fig. 1:**
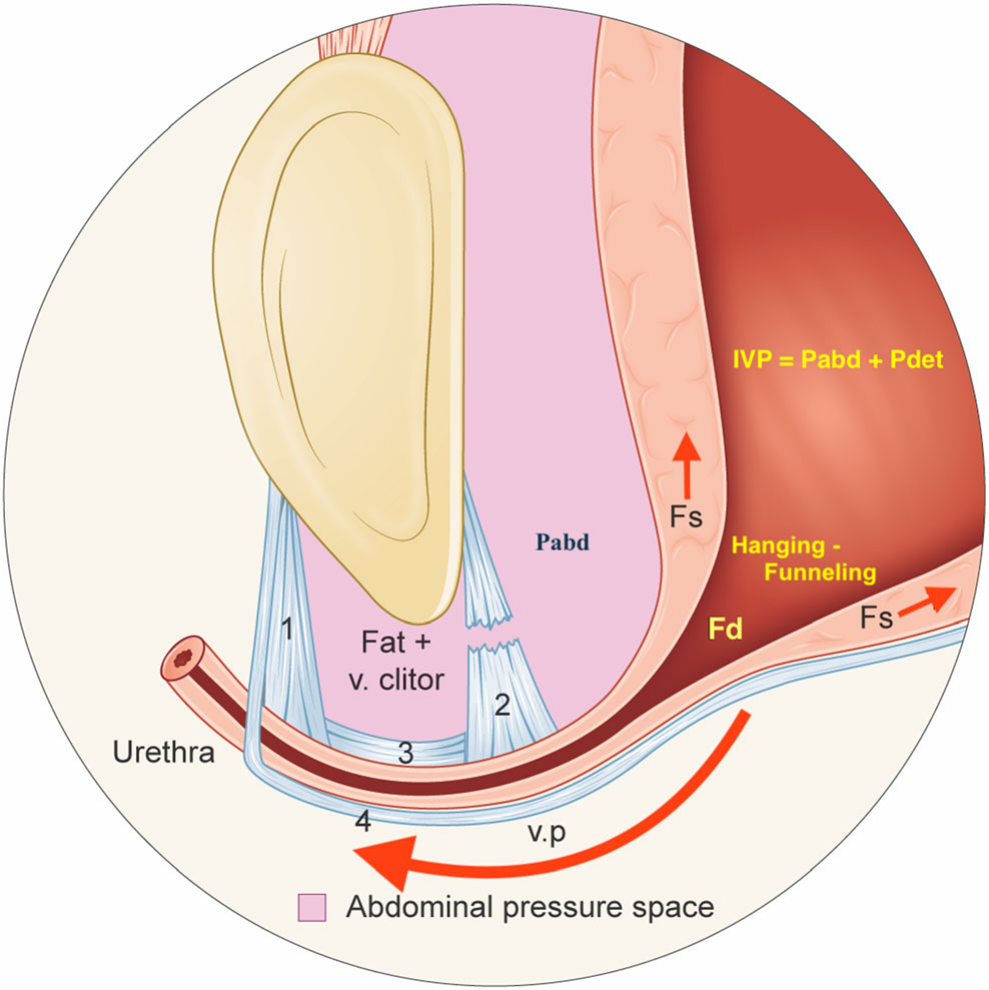
Illustration of hypermobile stress urinary incontinence during a Valsalva maneuver. The abdominal pressure space is indicated by the purple area, which comprises the proximal two-thirds of the urethra and bladder. The posterior pubourethral ligaments (PUL) are defective. The long urethra (4.5 cm) is “wheeling” downward hanging between the anterior PUL and the less mobile bladder neck. This generates a pulling force (Fs) that shears open/funnels the proximal urethra. In the illustrated case the Pabd is less than the abdominal leak point pressure and thus there is funneling without urine leakage. According to Pascal’s formula, the outflow distending force Fd = (Pdet+Pabd)*π*r*sqrt(r^2^ + h^2^) = IVP*π*r*s, where Pabd = abdominal pressure, Pdet = detrusor pressure, r = radius of the meatus internus, h = height of the funnel (cone), s = length of the funnel slant, and IVP = intravesical pressure (Pdet + Pabd). The maximum urethral pressure during stress resists the distending force (Fd), preventing leakage of urine, but the enforced distension of the proximal urethra may provoke urgency and frequency symptoms [4]. Severe mixed urinary incontinence (MUI) is often hypomobile stress urinary incontinence (SUI) with hanging/forced funneling even at rest. The intermediate PUL are not attached to the os pubis. Between these ligaments and the os pubis, there is only fat and the vena clitoridis. Enhörning’s theory stipulates reinforcement of the tissues in the anterior vagina, which yield during stress. The integral theory initially stipulated a suburethral tape starting 0.5 cm from the meatus externus., which was later changed to 1.0 cm, and still later to a mid-urethral position (this “restores the fulcrum”). The urethral hanging theory stipulates a suburethral tape starting 1 cm from the bladder neck, with the center of the tape at the vaginal point (v.p.) (this “stops urethral hanging”). This image can alternatively be interpreted to demonstrate a urethra with minimal mobility (“fixed urethra”), exhibiting hanging/funneling even at rest. In such a severely hypomobile SUI, a suburethral tension-free tape is of marginal, if any, benefit to the woman. To prevent hanging, the proximal urethra at the vaginal point (v.p.) must be lifted above its resting position. Lifting is also required in the case of a less hypomobile urethra which is not hanging at rest. This is because the use of tension-free vaginal tape (TVT) or transobturator tape (TOT) is associated with low cure rates as the downward distance for the urethra to reach a hanging position is short, and a high Pabd makes the TVT and TOT sway downward a little owing to their elasticity. A TOT, in particular, sways downward because it is similar to a 5–8 cm long horizontal hammock. This is in contrast to a TVT, which forms a tight vertical loop which is short because it is postoperatively adhered to the lower part of the pubic body. To create a lift without the risk of obstruction, the “TVT technique” can be employed to insert one tuned tape in the paraurethral tissue on each side of the v.p. or alternatively to elevate the proximal urethra by broadly folding the pubocervical fascia at the v.p. and then support the plicated fascia with a tension-free suburethral tape (TVT). 1, right anterior pubourethral ligament; 2, right posterior pubourethral ligament; 3, right intermediate pubourethral ligament; 4, pubocervical fascia; Fd, outflow distending force; Fs, shearing force; v. clitor, vena clitoridis, v.p., vaginal point (which corresponds to the attachment point of the posterior pubourethral ligaments to the pubocervical fascia on each side of the urethra)

The development of a new theory starts with an idea. The basic—and only—new idea relating to the urethral hanging theory (UHT) [1–5] is that, in the case of stress urinary incontinence (SUI), the proximal urethra descends to a hanging position on a less mobile bladder neck and is funneled. The rest of the theory builds on the works of others and the laws of physics. While some may call this speculation, I consider it “standing on the shoulders of giants.” Figure 1 shows the mechanism underlying urethral hanging and illustrates how this generates the forces that open the bladder neck and proximal urethra resulting in “forced funneling.”

In the 6th edition of the International Continence Society Book (2017), the authors state: “Many patients with urodynamic stress incontinence show urethral mobility, though it is not yet known what it is about that mobility which permits urethral opening during stress.” I propose that the UHT offers an answer to this enigma.

A video produced by the Sydney Pelvic Floor Health Clinic at the University of Sydney, Australia, provides an excellent demonstration of urethral funneling during a Valsalva maneuver in a woman with SUI. In my opinion and according to the UHT, this video shows that the anterior vaginal wall is pressed downward and underneath the os pubis, and—while the urethra is tethered to a less mobile bladder neck—it will draw the bladder downward into a tent formation before the proximal urethra is pulled open/funneled by shearing and distending forces (Fs and Fd). This video is available online [6] (see also Fig. 1).

In a recently published editorial, Abendstein expressed concerns that "many authors try to resuscitate Enhörning’s abdominal pressure transmission theory for the urethral closure" [7]. In this editorial, Abendstein cited one of my articles about the UHT [1].

The abdominal pressure transmission theory proposed by Enhörning (Enhörning’s theory, ET) is often misunderstood. It is regularly interpreted to mean that, in cases of SUI, the bladder neck descends outside the abdominal cavity, and treatment must involve elevating or repositioning the bladder neck. However, this actually contradicts the information provided in Enhörning’s original paper [8] and in his 1992 review article of the same paper [9]; ET should not be rejected although it does contain some errors.

According to ET, the urethra is divided into an inner proximal intraabdominal portion and an outer distal extraabdominal portion, above and below the urogenital diaphragm, respectively. The former is the larger of the two, comprising two-thirds of the total length of the urethra. Following this, ET involves two presumptions. One is that the proximal urethra is positioned inside the abdominal cavity and, consequently, within the “pressure equalization zone.” This implies that an increase in abdominal pressure (Pabd) is simultaneously “transmitted” in full to the bladder and proximal urethra. The second presumption is that, in cases of SUI, Pabd is not fully transmitted to the proximal urethra because of deficiencies in vaginal support and/or the intrinsic sphincter.

Enhörning articulated this as follows: “The deficient transmission may be due to a variable combination of two factors: (i) weakening of the circular or spiral muscles of the intrapelvic urethra, so as to widen this part into a funnel, and (ii) reduced power of the tissues in the anterior vagina to counteract sharp rises in intraabdominal pressure.” Enhörning also writes: “During cough, when the intraabdominal pressure increases, this tissue yields and is therefore unable to offer all the counterpressure required. More proximal tissues will be stretched and thereby made to offer part of the counterpressure. Thus, the increased intraabdominal pressure will not be completely transmitted to the internal portion of the urethra. The beneficial effect of surgical treatment for SUI may be attributed chiefly to a reinforcement of these tissues so that the urethra is more readily exposed to increases in intraabdominal pressure” [8].

When stating that “more proximal tissues will be stretched,” it is likely that Enhörning was explaining that when the suburethral vagina/urethra yields, part of the counterpressure will be accommodated by the vaginal support underneath the bladder. This notion is similar to the UHT, although Enhörning did not acknowledge that the urethra will yield, resulting in a hanging/forced-funneling situation where the shearing force (Fs) and outflow distending force (Fd) can counterbalance the fully transmitted Pabd. Instead, Enhörning presumed that—when the suburethral vagina yields without reaching a firm backstop—the counterpressure is partially proximally relocated to the vaginal wall below the bladder, resulting in a buffering effect (i.e., a damping “shock-absorber” effect) in response to the transmission of pressure to the proximal urethra.

However, this damping effect is not supported by the laws of physics; in particular, Pascal’s law of fluid pressures, which states that when there is an increase/change in pressure at any point of a confined fluid, there will be an equal increase/change at every other point in the container. Therefore, because the bladder and proximal urethra remain within the same closed “water bag” entity (the abdominal cavity), any change in Pabd will affect the whole entity and thus the bladder and proximal urethra will be equally affected.

Enhörning presumed that when the intravesical pressure exceeds the urethral pressure, the m.i. is pushed open. This presumption is inaccurate. According to the law of elastic collision, a molecule bouncing against the bladder wall would generate a force perpendicular to it. Therefore, a urethra that yields without reaching a hanging position on a less mobile bladder neck will not generate a pulling force. Concisely, a closed m.i. forms a perfect seal, which cannot be pushed open, it has to be pulled open; the intravesical pressure (IVP) is independent of the conditions behind a closed m.i., and accordingly the size of the urethral pressure is irrelevant for opening of the m.i.. however it is important for the secondary closure mechanism. The m.i. is the primary closure mechanism and the flow-controlling zone.

The integral theory (IT) postulates that, during stress, the levator plate and conjoined longitudinal anal muscles contract simultaneously, pulling the anterior vaginal wall down like a trapdoor, thereby shearing the posterior urethral wall from the better supported anterior urethral wall.

**Fig. 2:**
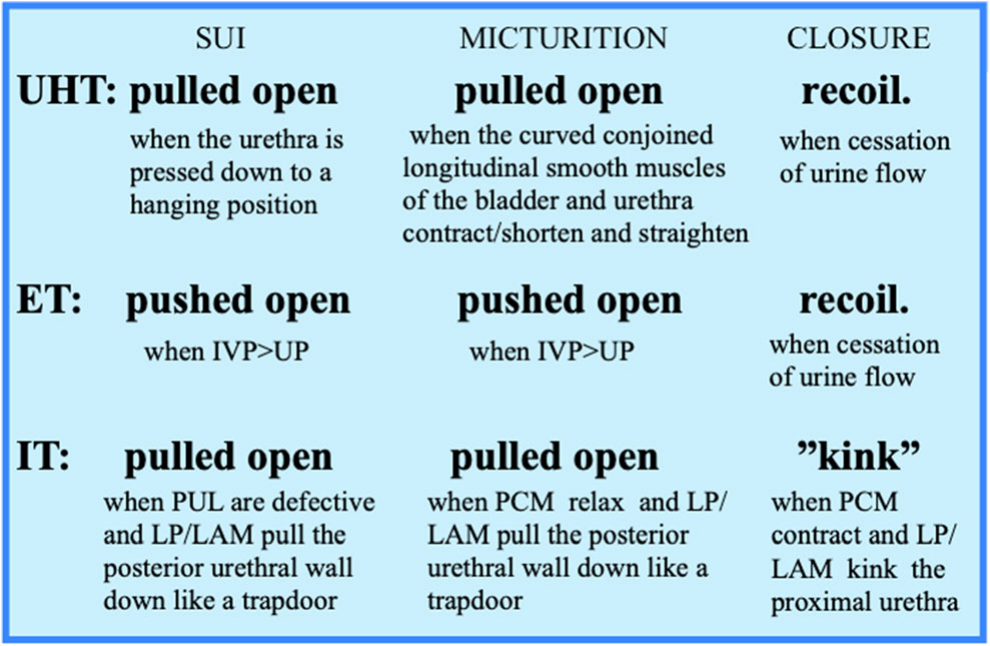
Bladder neck opening and closure during stress urinary incontinence and normal micturition. Differences between theories. IVP, intravesical pressure; UP, urethral pressure; PUL, posterior pubourethral ligaments; PCM, pubococcygeus muscles; LP, levator plate; LAM, longitudinal anal muscles

The three theories described here all postulate that SUI is a result of deficient posterior pubourethral ligaments (PUL), but they differ in their explanations of how this deficiency leads to urethral opening/funneling. The proximal urethra is described as being pulled open by urethral hanging on a less mobile bladder neck (UHT), pushed open when intravesical pressure exceeds urethral pressure (ET), or pulled open by contraction of the pelvic muscles (IT) (Fig. 2).

**Fig. 3:**
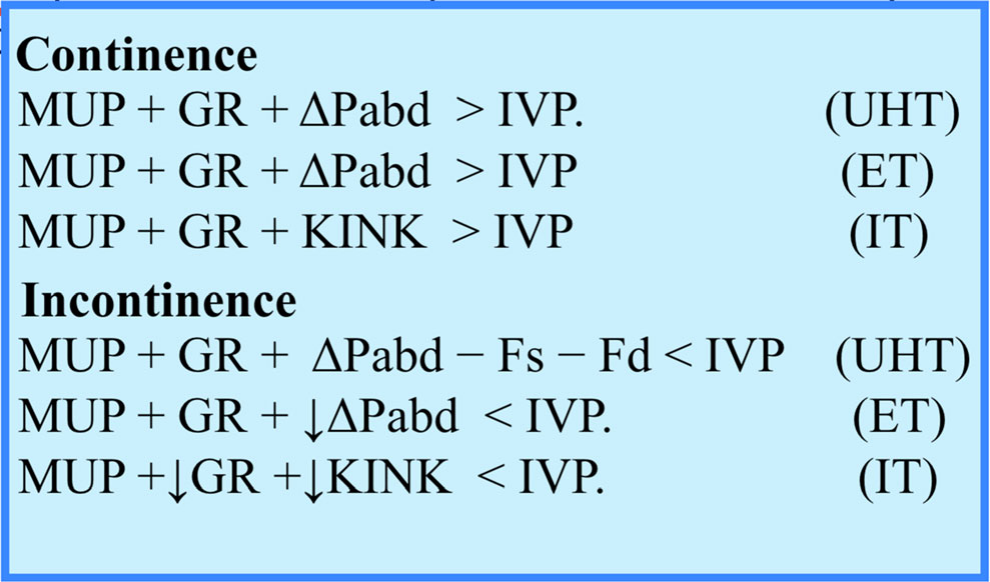
Definitions: MUP is the maximum urethral pressure at rest, GR is the guarding reflex (produced by the rhabdosphincter plus the pubococcygeus muscles, which lift the vaginal hammock and press the posterior urethra wall against its anterior wall), ΔPabd is the increase in the abdominal pressure during stress, ↓ΔPabd is the ΔPabd that is transmitted but reduced because of the damping effect, IVP is the intravesical pressure (detrusor pressure + Pabd), KINK represents the kinking/stretching/narrowing of the proximal urethra against the pubourethral ligament as a fulcrum, Fs is the shearing force, Fd is the outflow distending force, and ↓KINK represents the change in kinking due to loose and lengthened posterior pubourethral ligaments, which results in less stretching/narrowing of the proximal urethra and a reduction in the guarding reflex (↓GR) (according to the integral theory)

Continence and incontinence are defined according to the UHT, ET, and IT by the equations shown in Fig. 3.

According to the UHT and ET, the proximal urethra recoils to achieve closure after cessation of urine flow through the actions of its intrinsic muscles, vascular plexus, and the high content of collagen and elastic fibers. Concurrently, the levator ani muscles transition from relaxation to the normal high resting tonus, which elevates the pubocervical fascia/vaginal wall. The posterior PUL extend into the antero- medial part of the levator ani muscles—the pubococcygeus muscles—and are concomitantly elevated, thereby sustaining the correct spatial relationship between the proximal urethra and bladder neck, preventing urethral hanging as in UHT or pressure “damping” as described in ET. Transmission of Pabd to the proximal urethra occurs even at rest and is thus part of the secondary closure mechanism of the urethra. This secondary mechanism is important for voluntary interruption of flow, emptying the urethra after micturition, and obstructing retrograde flow during activities such as swimming.

According to the IT, closure occurs via a musculo-elastic mechanism. The distal urethra is closed when the “hammock” part of the anterior vaginal wall is pulled upwards by forward contraction of the anterior pubococcygeus muscles (referred to as the “first closure mechanism”). The proximal urethra is then closed by contraction of the two backward/downward-directed muscles, which kink/stretch to narrow the proximal urethra using the PUL as a fulcrum (referred to as the “second closure mechanism”). Closure also occurs via a “ball-valve” mechanism, wherein the bladder rotates around the insertion point of the pubovesical ligament (the arch of Gilvernet) to close the bladder neck [10, 11].

During normal micturition, it is thought that the m.i. is opened through various mechanisms (Fig. 2): pulled open when the curved conjoined inner longitudinal smooth muscles of the bladder and urethra—which are innervated by parasympathetic nerves — contract/shorten and straighten (as in UHT) [5], pushed open when the intravesical pressure exceeds the urethral pressure (as described in ET), or pulled open when the pubococcygeus muscles relax and the levator plate and conjoined longitudinal anal muscles contract, pulling the vaginal walls down and shearing the posterior urethral wall from the better supported anterior urethral wall (as in the IT). According to the UHT and IT, a closed m.i. provides a perfect seal that cannot be pushed open by high intravesical pressure.

The originators of the IT claim to be able to disprove ET. In the abovementioned editorial, Abendstein cited three studies supporting this suggestion [7]. In addition to these, I cite one study here. These four studies, which allegedly disprove ET, are as follows:

1. In 1990, the originators of the IT reported that 30/30 women were cured of SUI with no evidence of bladder elevation [12].

My comment: Enhörning does not stipulate elevation; ET stipulates reinforcement of the suburethral tissues so that the urethra does not yield downwards.

2. In a 1995 study, the originators of the IT showed that the pressures inside the urethra during coughing are greater than the pressures outside the urethra [13].

My comment: This is in agreement with ET, whereby MUP + GR + ΔPabd > Pabd. In this study, pressures were measured in equivalent positions, inside and just outside the urethral wall. The summation of pressure effects caused by the “transmitted” Pabd, the guarding reflexes, and the urethral pressures at rest cause the urethral pressures inside the urethra to exceed the external pressures (Pabd).

3. A 2009 rat study by Kamo et al., reported that, during sneezing, “the middle urethral closing response was observed and remained intact even after opening the abdomen” [14].

My comment: This is in agreement with ET. The guarding reflex still functions in the case of an opened abdomen, explaining the observed mid-urethral pressure increase during sneezing.

4. In 2003, Petros reported a study using a virtual operating technique [15], which contradicted ET (virtual operation: the recreation of a competent PUL through unilateral midurethral support using a forceps—a "simulated" operation).

My comment: Analysis of this study indicated that the apparent disproval of ET was incorrect because of a misinterpretation of the pressure measurements [5].

The originators of the IT claim they validated a central principle of the IT, namely, that the urethra is opened and closed by three bidirectional pelvic muscles using the posterior PUL as a fulcrum. In a study group that comprised 20 women with SUI, video-radiological studies were performed at rest, during stress, and at micturition, and electromyography (EMG) micturition studies were performed using a surface EMG probe placed in the posterior fornix of the vagina [16, 17]. The authors claim that, at micturition, radiographic images showed that the levator plate and conjoined longitudinal anal muscles pull down the posterior urethral wall and open out the urethra and that EMG signals show that these muscle actions commence well before micturition starts. Thus, the proximal urethra is actively opened prior to detrusor contraction. The same striated pelvic muscle mechanism is said to open out/funnel the proximal urethra in cases of SUI.

My comment: The described validation has low evidence value because surface EMG has many shortcomings. A review article published in 2017 [18], concerning surface EMG using intravaginal probes, questions the validity of this technique because of "crosstalk problems," meaning that recorded surface EMG activity may originate from neighboring muscles rather than coming exclusively from the muscles being investigated. There are also artifacts caused by patient movements and electrical devices in the room. The authors conclude that "Definitive conclusions are regularly drawn from an insufficient basis of evidence…. It remains unclear whether this issue can be solved at all with current (2017) technology.” Using early technology as in the cited study (1997) will likely be even more inaccurate. Using radiography, it is difficult to discriminate among a structure being plunged down, pushed down, or pulled down.

It has been shown that review of ultrasound analyses of urethrovesical mobility during coughing by an expert panel resulted in the correct identification of women with stress incontinence 57% of the time [19, 20], which is only 7% better than would be expected by chance alone. The authors concluded that this was “confirming that urethrovesical mobility is not strongly associated with stress incontinence” [20]. To improve accuracy, the experts should have attempted to evaluate urethral mobility in relation to the bladder neck. According to the UHT, urethral hanging on a less mobile bladder neck is indicative of SUI, and backstop before hanging is indicative of the absence of SUI. In a 2010 study reported by Pirpiris et al., which examined the correlation between segmental urethral mobility and symptoms and urodynamic findings, the authors found that SUI was strongly associated with midurethral mobility rather than bladder-neck mobility. The most significant association (*P* = 0.006) was related to a point 16 mm from the bladder neck [21]. This is in agreement with the UHT, which stipulates a suburethral tape starting 1 cm from the bladder neck, which is the center of tape at the vaginal point (v.p.), 15.5 mm from the bladder neck.

A prerequisite for urethral hanging is that the proximal urethra descends more than the bladder neck. If the proximal urethra and bladder neck descend to a similar extent, the urethra will not adopt a hanging position. The mobility of the proximal urethra in relation to the bladder neck determines whether SUI occurs; the descent of the proximal urethra is not important if the bladder neck descends correspondingly. It has long been known that the emergence of a cystocele can lessen or eliminate existing SUI as the urethra is prevented from hanging on the bladder neck. However, the opposite also applies; that is, surgical overcorrection of a cystocele can cause SUI. The posterior urethrovesical angle increases when the proximal urethra descends more than the bladder neck.

In an article published in 1993 [22], the originators of the IT unmistakably reveal that they have misinterpreted ET. In this publication, a radiograph showing the bladder neck position at rest and during strain is presented with the following legend: "If the intraabdominal pressure equalization theory were to be correct, a midurethral cough transmission ratio of 91% such as was registered in Fig. 1 would be an impossibility, as virtually the whole urethra is situated below the pubic bone." However, the pubocervical fascia delimits the abdominal cavity, not the bony pelvis, and thus the proximal urethra is always inside the abdominal cavity (the pressure equalization zone). Enhörning does not consider that a low-lying urethra outside the pressure equalization zone is the cause of SUI.

From the viewpoint of the UHT, the following summary can be made: ET is incorrect regarding the lower limit of the abdominal cavity, which is not the urogenital diaphragm but the pubocervical fascia. ET does not acknowledge that a closed m.i. forms a perfect seal that cannot be pushed open or that a funneled proximal urethra at rest has a functional, not morphological, cause. However, the core of ET provides a correct explanation of the role of Pabd transmission, agrees with laws of physics (excluding the idea of damping or buffering), and is correct in concluding that suburethral support restores continence. However, similar to the IT, ET does not differentiate between hyper- and hypomobile SUI and, consequently, does not recognize that the cure for hypomobile SUI should include lifting the proximal urethra, at the vaginal point (v.p.), above its resting position [4].

The originators of the IT rejected ET to develop their new theory that was built on false premises and some misinterpretations of experimental data. The idea that urethral closing and opening are accomplished by three external, bidirectional striated pelvic muscles using the posterior PUL as a fulcrum is not plausible given that perfect intrinsic mechanisms exist (i.e., closing by recoiling and opening by contraction of the conjoined inner longitudinal smooth muscles of the bladder and urethra). The bladder-urethra complex constitutes one organ with inherent smooth muscle-related internal mechanisms for closing, opening, relaxing, and contracting. This raises questions regarding the need for striated pelvic muscles mediated by a vulnerable PUL for the closing and opening of the bladder neck. Following the principle of Occam’s razor, the UHT should be accepted before the IT. The UHT is a biomechanical model to explain the pathophysiology of SUI and MUI and accordingly how to repair a defective anatomy.
